# Acidic environment could modulate the interferon-γ expression: Implication on modulation of cancer and immune cells’ interactions

**DOI:** 10.2478/abm-2023-0047

**Published:** 2023-09-17

**Authors:** Vishal Sharma, Jagdeep Kaur

**Affiliations:** Department of Biotechnology, Panjab University, Chandigarh 160014, India

**Keywords:** immune escape, interferon, macrophages, pH, T cells

## Abstract

**Background:**

In rapidly growing solid tumors, insufficient vascularization and poor oxygen supply result in an acidic tumor microenvironment, which can alter immune response.

**Objective:**

To investigate the role of the acidic microenvironment in immune response modulation along with cancer and immune cells’ interactions.

**Method:**

To mimic the tumor microenvironment conditions, T cells (Jurkat), macrophages (THP-1), and HeLa (cervical) cells were cultured under acidic conditions (pH 6.9, pH 6.5) and physiological pH (7.4). The HeLa cell culture medium was exploited as a tumor cell conditioned medium. Real-time PCR was carried out to quantify the mRNA levels, while flow cytometry and western blot hybridization was carried out to ascertain the levels of different proteins.

**Results:**

The acidic microenvironment around the T cells (Jurkat) and macrophage cells (THP-1) could lead to the downregulation of the interferon gamma (IFN-γ). An increase in IFN-γ expression was observed when Jurkat and macrophage cells were cultured in HeLa cells conditioned medium (HCM) at low pH (pH 6.9, pH 6.5). The HeLa cells under acidic environment (pH 6.9, pH 6.5) upregulated interleukin 18 levels and secreted it as exosome anchored. Additionally, enhanced nuclear localization of NF-κB was observed in Jurkat and THP-1 cells cultured in HCM (pH 6.9, pH 6.5). Jurkat and THP-1 cultured in HCM revealed enhanced cytotoxicity against the HeLa cells upon reverting the pH of the medium from acidic to physiological pH (pH 7.4).

**Conclusion:**

Collectively, these results suggest that the acidic microenvironment acted as a key barrier to cancer and immune cells’ interactions.

The potential of tumors to grow and metastasize is the consequence of not only oncogenic mutations but also the behavior of non-malignant cells in the tumor microenvironment [1]. Tumor cell metabolism, aerobic glycolysis, and production of lactate and H^+^ ions resulted in the acidic microenvironment [2]. Cancer cells’ distinct glycolytic metabolism generated an excessive level of lactic acid [3]. Thus, acidosis in the tumor microenvironment might result from the export of protons and lactic acid from tumor cells into the extracellular space by acid-base regulators like Na+/H+ exchangers and monocarboxylate transporters [4]. The pH of blood and tissue is closely regulated at a pH of 7.4 under normal physiological circumstances while the tumor microenvironment frequently had a pH range of 5.5–7.0 [5, 6]. Additionally, tumor cells intracellular pH is slightly alkaline compared to the external environment, which was reported to promote cell tumor progression [7]. Both T cells and macrophages were considered as the most important defense players against pathogens and cancer cells, respectively[8].

While nascent transformed cells could be eradicated by an innate immune response at an early stage, tumor progression could be targeted by an adaptive immune response motivated by antigen-specific T cells [9]. The anticancer immune response, associated with effector T cells, was documented to be extremely reliant on components of the microenvironment such as helper cells and cytokines [10]. However, the functions of various components are also influenced by environmental pH. Acidic pH could significantly hinder the function of standard immune cells. Furthermore, tumor-derived soluble factors facilitated the escape of cancer cells from immune surveillance, allowing tumor development and metastasis [11]. Failure of the immune system to detect and target transformed cells has already been reported in cancer development and progression [12]. Recently, macrophages were demonstrated to support cancer cells by secreting some chemokines [13]. Tumor cells might avoid T cell attack through a variety of mechanisms. However, it is unclear what causes cancer cells to evade immune response or immune clearance under the prevalence of acidic pH. The major populations of cells that infiltrate developing cancer include immune cells, fibroblasts, adipocytes, endothelial cells, and perivascular cells [14].

The T cells infiltrate different parts of a tumor differently depending on what extracellular matrix components were posing a hindrance to T cell motility. Additionally, there are different reports showing that T cell infiltration in the tumor is associated with better prognosis. This is well documented in a number of studies [15,16,17]. Keeping in mind the acidic microenvironment, the present study was designed to investigate the effect on interaction of cancer and immune cells i.e. T cells and macrophages. T cells and macrophages are the primary producers of interferon gamma (IFN-γ), one of the major immune-modulatory molecules, which directs various cellular events via transcriptional regulation of immunologically relevant genes [18]. IFN-γ has been shown to play an important role in the majority of immunological responses, including cell-mediated immunity and inflammatory responses [19]. It has been documented for its direct effect on tumor cells and was reported to play a major role in tumor cell eradication [20]. Interleukin 18 (IL-18) has been documented as an IFN-γ inducing factor [21]. The activation of IL-18 and its export have been demonstrated in humans and mice to target the immune cells and provoke IFN-γ production. Additionally, IFN-γ also played a role in antitumor immunity [22]. Therefore, IFN-γ has been considered as a major immune-modulatory molecule in the present study.

Furthermore, an acidic tumor microenvironment might reduce the efficacy of anticancer therapy [23].

Acidic pH has been recently demonstrated to induce the octamer-binding transcription factor 4, which further modulated the recruitment and reprograming of tumor-associated stromal cells and had an impact on the recruitment and activity of immune cells [24, 25]. Apart from tumor cells, the behavior of immune cells and their ability to invade the tumor were also dependent on the microenvironment as an intermediate medium. Therefore, we considered HeLa cells conditioned medium (HCM) under acidic pH as the tumor microenvironment in our study. The present investigation examined the effect of the acidic microenvironment generated by cancer cells on immune response in terms of expression of IFN-γ and IL-18. Further, attempts were also made to ascertain the role of the acidic microenvironment in modulating immune and cancer cells’ interactions.

## Methods

### Cell culture and treatment with acidic medium

Exponentially growing HeLa cells (human cervical cancer), Jurkat cells (human T cell lymphoma), and THP-1 cells (monocytes) were procured from the National Centre for Cell Science (NCCS), India. THP-1 (monocytes) were differentiated to macrophages by using PMA (5 ng/mL) for 48 h [26]. HeLa cells were allowed to adhere for 48 h before being exposed to experimental conditions. The cells were cultured at 37°C under humidified 5% CO_2_ and a 95% air atmosphere. The cell density was maintained at a level lesser than 3 × 10^5^ cells/mL in 25 cm^2^ plastic tissue culture flasks with 10 mL of RPMI-1640. The culture medium was supplemented with 10% fetal bovine serum (Hi Media). LPS-free reagents were used during the experiments. The pH of the culture media was adjusted using 1N HCl. The experiments were carried out in the exosome-free fetal bovine serum [27].

**Figure 1.**
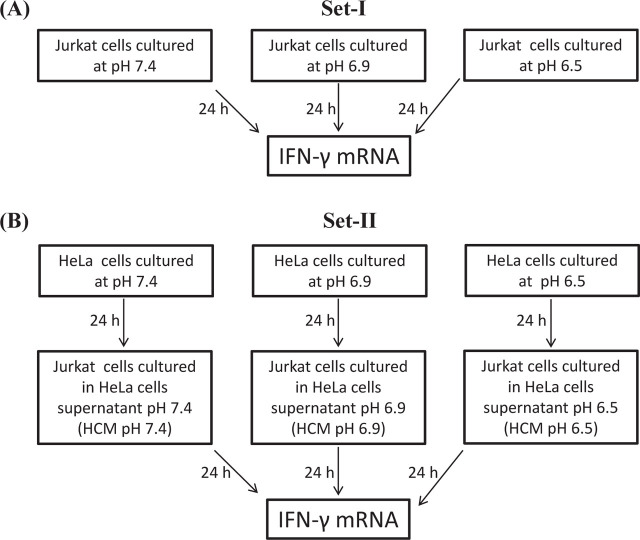
Schematic representation of experimental set up. **(A)** To mimic the effect of acidic pH on immune cells, Jurkat cells were exposed to different pH conditions, pH 7.4, pH 6.9, and pH 6.5, for 24 h. Total mRNA was isolated for further experiments. **(B)** To ascertain the effect of cancer cells in the acidic environment on immune cells, Jurkat cells were cultured in HCM of pH 7.4, pH 6.9, and pH 6.5 for 24 h. Further, total mRNA was isolated from Jurkat cells for further experiments. Similar studies were performed for THP-1 also. HCM, HeLa cells conditioned medium.

To ascertain the effect of acidic pH on immune cells, Jurkat (T cells) and THP-1 (macrophages) cells were cultured in pH 7.4, pH 6.9, and pH 6.5 media (**Figure 1A**). Further, to ascertain how cancer cells in an acidic environment can influence immune response, HeLa cells were plated at a density of 0.5 × 10^6^/mL in media having pH values of 6.5, 6.9, and 7.4. A low cell density was maintained, avoiding the effect of media starvation. After 24 h of incubation, the supernatant from HeLa cells was centrifuged at 2000 rpm for 5 min followed by filtration through a 0.22 μm membrane for further experimentation. This was referred to as HCM (**Figure 1B**). Further, Jurkat and THP-1 cells were plated at the density of 0.5 × 10^6^/mL in HCM of pH 7.4, pH 6.9, and pH 6.5 for 24 h (**Figure 1B**). The experiment was not extended beyond 24 h and at a pH lower than 6.5 since both these conditions have been shown to induce apoptosis in cells [28].

### Cytotoxicity assay

The cytotoxic effect of acidic pH was studied on Jurkat, HeLa, and macrophages cells by the MTT (3-(4,5-dimethylthiazol-2-yl)-2,5-diphenyl tetrazolium bromide) assay [29]. Briefly, the cells were cultured in 96 well plates at a density of 1 × 10^4^ cells per well at different pH values (7.4, 6.9, and 6.5). After incubation for 24 h, MTT (5 mg/mL in PBS) was added to each well and further incubation was carried out at 37°C for 2 h. Dark blue formazan crystals formed were dissolved in DMSO and optical density was measured by a microplate reader (Bio-Rad) at a wavelength of 570 nm.

### Gene expression analysis

Isolation of total RNA from cells after exposing them to experimental conditions was done using TRIzol reagent (Sigma). First strand cDNA (complementary DNA) synthesis was carried out using the Verso cDNA kit (Thermo Scientific). DNA contamination in the isolated RNA was eliminated using the RT enhancer available with the kit. Semi-quantitative PCR was carried out and amplified PCR products were resolved by 2% agarose gel electrophoresis. Quantitative real-time PCR analysis was performed in a real-plex system (Eppendorf) using the SYBR Green PCR Master mix (Thermo Scientific). Real-time PCR was carried out for *IFN-*γ, *IL-10*, *IL-18*, and *β-actin* using gene specific primers, as mentioned in **Table 1**. β-actin served as an internal control. Melting curve analysis was performed to confirm the specificity of amplified products, and delta CT method was used to quantify the alteration in gene expression.

**Table 1.** Details of primers used in the study

**Gene**	**Primer sequence**
*IFN-*γ	5′ TCCCATGGGTTGTGTGTTTA 3′ (F) 5′ AAGCACCAGGCATGAAATCT 3′ (R)
*IL-18*	5′ GCCTAGAGGTATGGCTGTAA 3′(F) 5′ TTATCATGTCCTGGGACA 3′ (R)
*IL-10*	5′ AGGAGTCCTTGCTGGAGGA 3′ (F) 5′ AAAGGCATTCTTCACCTG 3′ (R)
*β-actin*	5′ GTGGGCCGCTCTAGGCACCA 3′ (F) 5′ GGTTGGCCTTAGGGTTCAGGGGGG 3′ (R)

β-actin, beta actin; F, forward; IFN-*γ*, interferon gamma; IL-10, interleukin 10; IL-18, interleukin 18; R, reverse.

### Flow cytometry based *IFN-γ* gene expression analysis

Both Jurkat and THP-1 cells (0.5 × 10^6^ cells/mL) were washed in PBS, then fixed and permeabilized using the Cytofix/Cytoperm kit (BD Bioscience) for 20 min on ice. Cells were pelleted, washed with perm wash buffer (BD Bioscience), and stained for 1 h at 4°C with a fluorochrome-conjugated anti–IFN-γ monoclonal antibody (BD Bioscience). Cells were washed twice with a perm wash buffer and finally re-suspended in a perm wash buffer for flow cytometry analysis. Flow cytometric analysis was performed on BD FACS Canto II (BD Biosciences) for maximum cell counts of 10,000 and analyzed using BD FACSDiva software.

### Exosome preparation for IL-18 expression analysis

After culturing of HeLa cells for 24 h at different pH, the supernatant was collected and subjected to the centrifugation steps of 400 × g (10 min) to remove the intact cells, 3000 × g (20 min) to remove the cell debris, and 10,000 × g (30 min) to remove micro vesicles. The exosomes were then pelleted at 64,000 × g (110 min) and 100,000 × g (sucrose gradient) for 1 h. Total amount and concentration of exosomal proteins in the pooled samples were measured by the BCA method using protein estimation kit (Bangalore Genei) and subjected to western blot analysis (IL-18, CD 63).

### Western blot analysis

Cells were harvested, washed with cold PBS, and lysed in lysis buffer (20 mM Tris-HCl, pH 7.5, 150 mM NaCl, 1% NP-40, 1 mM ethylene glycol-bis (b-aminoethyl ether)-tetraacetic acid, 1 mM EDTA, 50 mM NaF, 1 mM b-glycerophosphate, 2.5 mM sodium pyrophosphate, 1 mM orthovanadate, protease inhibitor cocktail, and 1 mM PMSF). Protein estimation was determined by the BCA method using a protein estimation kit (Bangalore Genei). An equal amount of cell lysate from different test samples was resolved on 10% SDS-PAGE followed by transfer onto a PVDF membrane. The blots were probed with (1:1000) anti NF-κB (Sigma), IL-18, p38 MAPK, and CD63. Horseradish peroxidase-conjugated secondary antibodies were used to detect immune reactive bands using 3,3′-diaminobenzidine as a substrate.

### Subcellular fractionation

After treatment, Jurkat and THP-1 cells were suspended in hypotonic buffer (250 mM sucrose, 20 mM Hepes [pH 7.5], 10 mM KCl, 1.5 mM MgCl_2_, 1 mM EDTA, 100 μM PMSF) on ice and incubated for 30 min, followed by lysing the cells by vigorous pipetting for 30 min. Cell lysate from the previous step was centrifuged at 3000 rpm for 5 min at 4°C to remove nuclei. The supernatant was saved as cytoplasmic fraction. The pellet was washed again and saved as nuclear fraction and further subjected to western blotting as described above.

### Cytotoxic effect assessment after reversal of pH

**Figure 2.**
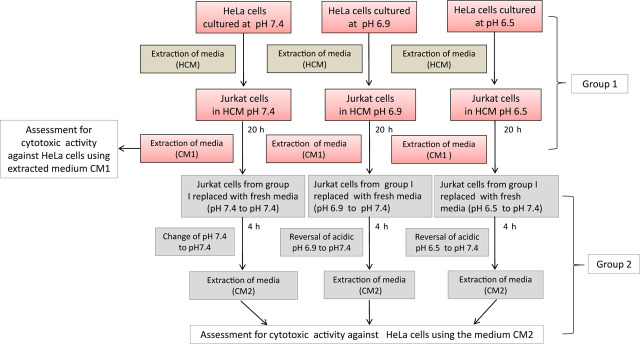
Schematic representation of experimental setup to ascertain the effect of the acidic environment in the restriction of immune response. Jurkat cells were stimulated with HCM at pH 7.4, pH 6.9, and pH 6.5 for 20 h. Cell-free media thus obtained were referred to as CM1 and saved for cytotoxic assessment against the HeLa cells. Jurkat cells obtained from this step were further subjected to reversal of pH by culturing the cells in a fresh medium of pH 7.4 for 4 h. After this, the media were extracted, referred to as CM2, and saved for cytotoxic assessment against the HeLa cells. A similar experiment was also carried out for THP-1. HCM, HeLa cells conditioned medium.

To determine the role of acidic environment (as an intermediate medium between cancer cells and immune cells) in the modulation of immune and cancer cells’ interactions, Jurkat or THP-1 cells were cultured in HCM of pH 6.5, pH 6.9, and pH 7.4 for 20 h and the obtained cell-free medium (CM1) was saved for cytotoxicity assessment (**Figure 2**). Further, the cells thus harvested were then cultured in fresh medium at pH 7.4 for 4 h and cell-free culture medium (CM2) was again saved for cytotoxicity assessment (reversal of acidic pH 6.9, pH 6.5 to physiological pH 7.4). HeLa cells were then cultured in CM1 and CM2 separately for each pH condition and viability was assessed by MTT assay to examine the cytotoxic effect of biological factors secreted by Jurkat cells, macrophages and modulated by different pH conditions and after reversal of pH to 7.4 (**Figure 2**).

### Statistical analysis

The needed statistical procedures were applied using SPSS 13.0, and data are presented as mean ± standard error of mean (SEM). One-way ANOVA (Analysis of variance) was used to analyze the results and to determine the significance of the mean between groups. *P* values <0.05 were considered significant.

## Results

The microenvironment around the tumor was reported to be acidic and plays a major role in cellular response [30]. Many cellular responses such as cytosolic and membrane-associated enzyme activities, ion transport activity, DNA synthesis, cAMP, and calcium levels are affected by acidic extracellular pH [31, 32]. Indeed, the tumor–stroma interface at tumor sites under the acidic microenvironment might have a diverse effect on tumor survival, outgrowth, organ homing, and invasion [33].

### Effect of acidic pH on the viability of HeLa, Jurkat, and THP-1 cells

Before initiating the experimental study, the effect of acidic pH on the viability of cells was examined. The viability of HeLa and THP-1 cells did not differ significantly (*P* = 0.3281), whereas Jurkat cells showed a nearly 10% decrease in viability at pH 6.5 when compared with cells cultured at pH 7.4 (*P* = 0.04).

### IFN-γ expression was downregulated in acidic environment in Jurkat and THP-1 cells

**Figure 3.**
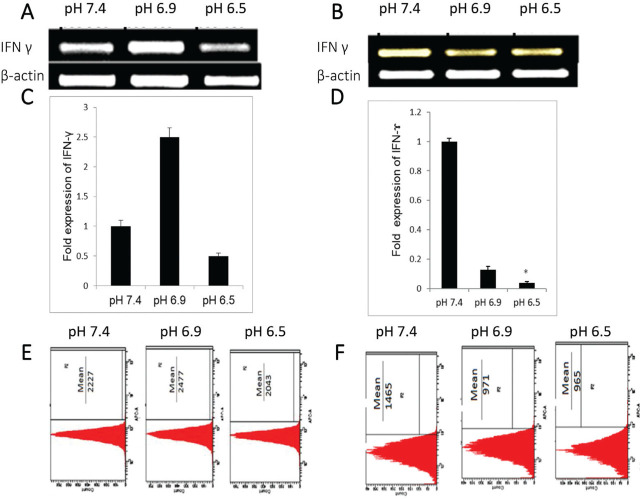
Expression of IFN-γ under physiological pH (pH 7.4) and acidic environment (pH 6.9, pH 6.5) in Jurkat **(A, C, E)** and THP-1 **(B, D, F)** cells after 24 h incubation. **(A, B)** Semi-quantitative PCR of IFN-γ mRNA expression under physiological pH (pH 7.4) and acidic environment (pH 6.9, pH 6.5). Representative agarose gel of PCR amplified products from 1 of 3 independent similar experiments. (**C, D**) Quantitative real-time PCR analysis of IFN-γ mRNA expression under physiological pH (pH 7.4) and acidic environment (pH 6.9, pH 6.5). The delta CT method was used for relative quantification. For relative quantification, cells grown at pH 7.4 were taken as control. Data were normalized to β-actin. The specificity of reaction products was analyzed by melting curve analysis. The bar graph indicated the fold change in the expression of IFN-γ. **(E, F)** Representative histogram of flow cytometric analysis of anti–IFN-γ antibody stained Jurkat cells (**E**) and THP-1 cells (**F**). Mean fluorescence depicted the level of IFN-γ. Ten thousand events were counted per tube. Data are representative of 1 of 3 independent similar experiments. *P < 0.05. IFN-γ, interferon gamma.

Upon exposure of Jurkat cells to acidic conditions (pH 6.5), the expression of IFN-γ mRNA declined in comparison with Jurkat cells exposed to pH 7.4 (**Figures 3A and 3C**). On the other hand, Jurkat cells cultured in pH 6.9 medium demonstrated upregulation of IFN-γ mRNA by 2.3 fold when compared with control (pH 7.4) (**Figures 3A and 3C**) (*P* = 0.22). These results were further supported by flow cytometric analysis (**Figure 3E**). Jurkat cells cultured at physiological pH 7.4 and stained with fluorochrome-conjugated anti–IFN-γ antibody had a mean fluorescence of 2227 whereas cells cultured at pH 6.9 and pH 6.5 had mean fluorescence of 2477 and 2043, respectively (**Figure 3E**). Culturing of differentiated THP-1 cells in the acidic microenvironment could have resulted in a decrease in IFN-γ expression at both pH 6.5 (*P* = 0.022) and pH 6.9 (*P* = 0.41) (**Figures 3B and 3D**). Flow cytometric analysis of fluorochrome-conjugated anti–IFN-γ antibody stained THP-1 cells cultured at physiological pH 7.4 revealed a mean fluorescence of 1465, while cells cultured at pH 6.9 and pH 6.5 revealed mean fluorescence of 971 and 965, respectively (**Figure 3F**). Collectively, this indicated a restriction in IFN-γ expression in both macrophages and T cells in the acidic microenvironment, which may play a role in thwarting the immune response.

### HCM-stimulated IFN-γ expression in Jurkat and THP-1 cells in the acidic environment

**Figure 4.**
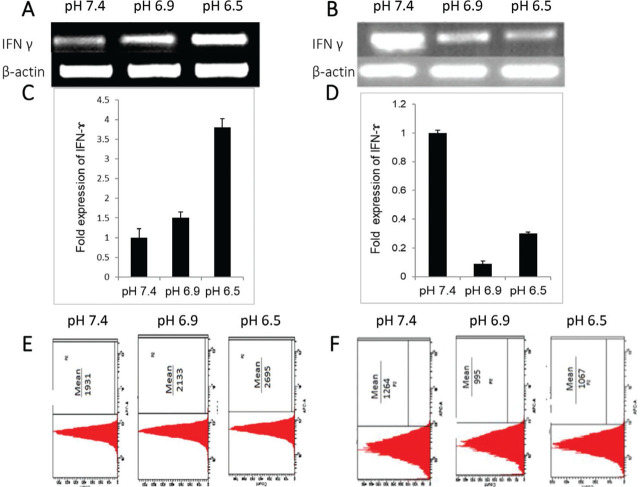
Expression of IFN-γ in Jurkat **(A, C, E)** and THP-1 **(B, D, F)** cells cultured in HCM at different pH (pH 7.4, pH 6.9, and pH 6.5). **(A, B)** Semi-quantitative PCR of IFN-γ mRNA expression in Jurkat and THP-1 cells cultured in HCM at pH 7.4, pH 6.9, and pH 6.5. Representative agarose gel of PCR amplified product from 1 of 3 independent similar experiments. **(C, D)** Quantitative real-time PCR analysis of altered IFN-γ mRNA level in Jurkat and THP-1 cells cultured in HCM at pH 7.4, pH 6.9, pH 6.5. For relative quantification, cells grown in HCM at pH 7.4 were taken as control. Data was normalized to β-actin. The specificity of the reaction products was analyzed by melting curve analysis **(B)** Representative histogram of flow cytometric analysis of IFN-γ stained Jurkat **(C)** and THP-1 **(D)** cells after treatment. Mean fluorescence depicted the level of IFN-γ. Ten thousand events were counted per tube. Data are representative of 1 of 3 independent similar experiments. HCM, HeLa cells conditioned medium; IFN-γ, interferon gamma.

**Figure 5.**
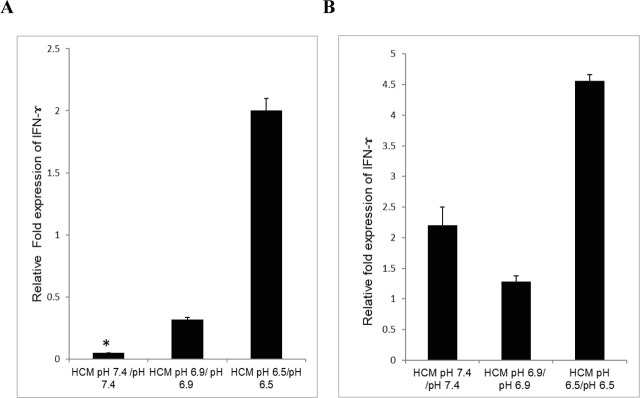
Altered relative expression of IFN-γ in Jurkat cells and THP-1 cells with and without stimulation with HCM at different pH values of 7.4, 6.9, and 6.5. A quantitative real-time PCR based comparison of IFN-γ produced by Jurkat cells **(A)** and THP-1 **(B)** cultured at different pH values (7.4, 6.9, and 6.5) and HCM at different pH (HCM pH 7.4, HCM pH 6.9, and HCM pH 6.5). *P < 0.05. HCM, HeLa cells conditioned medium; IFN-γ, interferon gamma.

To explore the role of factors secreted by cancer cells that help them to evade the immune response, Jurkat cells and macrophages were cultured in HCM. Jurkat cells cultured in HCM at pH 6.9 and pH 6.5 demonstrated 1.5 fold (*P* = 0.24) and 3.7 fold (*P* = 0.07) upregulation in levels of IFN-γ mRNA, respectively, than Jurkat cells cultured in HCM at pH 7.4 (**Figures 4A and 4C**). Flow cytometric analysis of fluorochrome-conjugated anti–IFN-γ antibody stained Jurkat cells cultured in HCM at pH 7.4 revealed a mean fluorescence of 1931, whereas cells cultured in HCM at pH 6.9 and pH 6.5 revealed mean fluorescence of 2133 and 2695, respectively (**Figure 4D and E**). These results suggest that the acidic microenvironment may be beneficial to IFN-γ expression. Following that, the level of IFN-γ mRNA in Jurkat cells cultured at pH 7.4 (without HCM) was significantly downregulated (8 fold) than in Jurkat cells cultured in HCM at pH 7.4 (**Figure 5A**) (*P* = 0.02). When Jurkat cells were cultured at pH 6.9 without and with HCM, there was a further downregulation (4 fold) in IFN-γ expression (**Figure 5A**) (*P* = 0.48). In contrast, when similar studies were performed at pH 6.5, IFN-γ mRNA was found to be upregulated (2 fold) (**Figure 5A**) (*P* = 0.29). Furthermore, when THP-1 cells were cultured in HCM, the level of IFN-γ was downregulated at pH 6.9 (*P* = 0.58) and pH 6.5 (*P* = 0.35) compared with pH 7.4 (**Figure 4B**). Similarly, flow cytometric analysis of THP-1 cells cultured in HCM at pH 7.4 revealed a mean fluorescence of 1264, while cells cultured in HCM at pH 6.9 and pH 6.5 revealed mean fluorescence of 995 and 1067 (**Figure 4F**). When THP-1 cells were cultured in media with pH 7.4, pH 6.9, and pH 6.5 and THP-1 cells were cultured in HCM with pH 7.4, pH 6.9, and pH 6.5 compared, upregulation of IFN-γ (4.5 fold) was observed at HCM pH 6.5 as compared to pH 6.5 (**Figure 5B**) (*P* = 0.065). RT PCR was used to examine IFN-γ expression in HeLa cells under different pH conditions (pH 7.4, pH 6.9, and pH 6.5). The results showed that IFN-γ was not expressed in HeLa cells at any pH.

### Under acidic environment, HeLa cells upregulated IL-18 and secrete exosome-anchored IL-18

**Figure 6.**
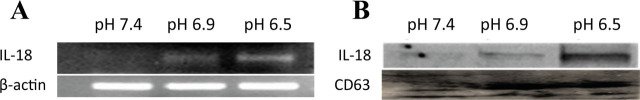
Effect of acidic microenvironment on the expression of IL-18 in HeLa cells. **(A)** Semi quantitative PCR analysis of IL-18 expression. epresentative agarose gel of PCR amplified product from 1 of 3 independent similar experiments. β-actin was used as an internal control. **(B)** Immunoblot analysis of IL-18 in exosome preparation from the culture medium of HeLa cells that were grown at different pH. Total exosome lysates were resolved on 10% SDS-PAGE for immunoblot analysis of IL-18 and CD63. CD63 was used as an exosome marker. IL-18, interleukin 18.

**Figure 7.**
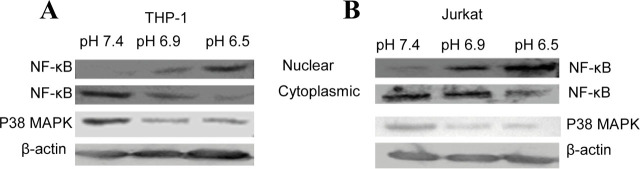
Western blot analysis of subcellular localization of NF-κB and expression of p38 MAPK in THP-1 **(A)** and Jurkat **(B)** cells cultured in HCM at the various pH values of 7.4, 6.9, and 6.5 for 24 h. Total cell lysate or cell lysate after fractionation (nuclear fraction and cytoplasmic fraction) were resolved on 10% SDS-PAGE for immunoblot analysis of NF-κB and p38 MAPK. β-actin was taken as an internal control. The data represent 1 of 3 similar experiments. HCM, HeLa cells conditioned medium.

The upregulation of IFN-γ in Jurkat and THP-1 cells cultured in HCM at acidic pH suggests that cancer cells may secrete biological factors to stimulate these cells. As a result, we examined the expression of IL-10 and IL-18, which have been shown to alter IFN-γ expression in various cells [34]. Total mRNA from HeLa cells (pH 7.4, pH 6.9, pH 6.5) was isolated and analyzed for the expression of IL-10 and IL-18 to investigate their role in IFN-γ expression. No expression of IL-10 was observed. However, enhanced expression of IL-18 was observed in HeLa cells cultured in a medium of acidic pH (**Figure 6A**). As a result, efforts were made to investigate the role of IL-18 in the acidic microenvironment and its relationship to IFN-γ expression. The acidic microenvironment around cancer cells stimulated the expression of IL-18, which in turn might be correlated with the IFN-γ mediated immune response. Further, western blot analysis of exosomes prepared from the cell-free supernatant of HeLa cells showed an association of IL-18 with the exosomes (**Figure 6B**). IL-18 was reported to modulate IFN-γ expression via NF-κB and MAPK kinase mediated pathways [35]. Therefore, the role of NF-κB and MAPK molecules in the modulation of IFN-γ were further investigated. At lower pH, HCM treated Jurkat and THP-1 cells showed a decrease in p38 MAPK expression (**Figure 7**). An increase in the level of NF-κB in the nuclear fraction was evident both in Jurkat cells and THP-1 cells cultured in HCM at lower pH. These results suggest the involvement of NF-κB in the modulation of IFN-γ. These findings suggest that under acidic conditions, HeLa cells secreted exosome-anchored IL-18, which might be correlated with IFN-γ expression.

### Cytotoxic effect assessment after reversal of pH

**Figure 8.**
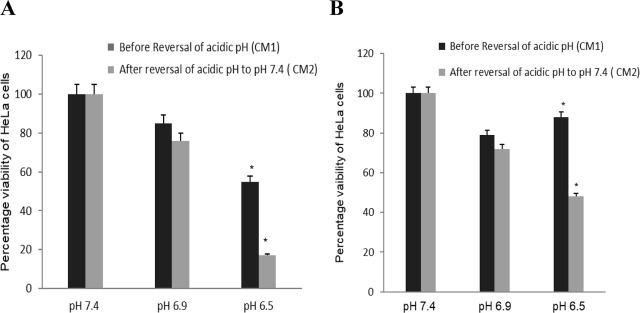
A comparison of the cytotoxic activity of THP-1 **(A)** and Jurkat **(B)** against the HeLa cells after reverting the pH (7.4, 6.9, 6.5) to physiological pH (7.4). Cytotoxic activity was assessed by MTT assay. *P < 0.05.

As previously stated, the acidic environment induced IL-18 correlated with IFN-γ upregulation in Jurkat and THP-1 cells. Therefore, attempts were made to decipher the behavior of HeLa cells in the culture medium taken from Jurkat and THP-1 cells grown in a medium of different pH. To investigate this, HeLa cells were cultured for 24 h in CM1 and CM2 (pH 7.4, pH 6.9, and pH 6.5) obtained from Jurkat cells to mimic the natural tumor conditions as described in the Methods section. HeLa cells showed 88% viability at pH 6.5 (**Figure 8B**) in CM1, whereas the viability was decreased to 48% in cells cultured in CM2 obtained from the growth of Jurkat cells (reversion of pH to 7.4) (*P* = 0.32). The viability of HeLa cells was determined similarly to the CM1 and CM2 obtained from THP-1 cells. At pH 6.5, only 55% of the HeLa cells were viable in CM1, while viability was reduced to 17% in cells cultured in CM2 (**Figure 8A**) (*P* = 0.04).

## Discussion

An acidic microenvironment around the tumor cells is a key feature of actively growing tumors. Immune cells are rarely in contact with cancer cells; instead, they occupy the interstitial acidic medium. Therefore, an attempt has been made in the present study to understand the mechanism by which the acidic microenvironment affected the tumor and immune cells’ interactions. The inhibition of acidic microenvironments by exploiting proton pump inhibitors has been widely studied [36, 37]. Recent studies revealed the chemosensitization of cancer cells that underwent proton pump inhibitor treatment [38, 39].

Jurkat and THP-1 were cultured in acidic pH media (pH 6.9, pH 6.5) and normal physiological pH (pH 7.4). The upregulation of IFN-γ following mild acidification of the medium to pH 6.9 around Jurkat cells suggests that mild acidification in the immune cell microenvironment might favor the IFN-γ mediated immune cell response. The downregulation of IFN-γ in response to pH 6.5, on the other hand, suggests that an acidic microenvironment can modify or restrict the IFN-γ mediated immune response. Similar research with THP-1 (macrophages) has suggested that acute acidification around immune cells could impair immune performance and reduce IFN-γ mediated immune response. These studies were in agreement with the previous studies where it was observed that the acidic environment generated by lactic acid could impair the expression of various cytokines and cytolytic activity, as well as cause anergy of T lymphocytes [40, 41]. The activity of natural killer (NK) cells, as well as lymphokine-activated killer (LAK) cells, has also been reported to be reduced along with the decreased release of TNF-α, IFN-γ, IL-10, IL-12, and transforming growth factor-1 (TGF-1) under acidic conditions [42, 43].

Furthermore, it was intriguing whether the interaction of cancer and immune cells would favor immune suppression or provoke the immune response in terms of IFN-γ in an acidic environment. These observations generated in the present study suggest that an acidic environment around cancer cells could enhance the IFN-γ mediated immune response by stimulating the immune cells. As a result, an acidic microenvironment might be beneficial in stimulating IFN-γ expression. Interestingly, a comparison between immune cells cultured in HCM (pH 7.4) to those without HCM (pH 7.4) suggests downregulation of IFN-γ. These findings suggest that under physiological pH (pH 7.4) conditions, cancer cells could secrete some unknown factors inhibiting IFN-γ expression by immune cells, there by helping cancer cells to evade immune response. This was in agreement with the previous studies where it had been shown that cancer cells secreted some unknown factors, which could lead to the suppression of cytokines [44]. When Jurkat and THP-1 cells were compared in HCM (pH 6.9, pH 6.5) and without HCM (pH 6.9, pH 6.5), IFN-γ was found to be upregulated. Under acidic conditions, it appeared that cancer cells secreted unknown biological factors that cause immune cells to secrete IFN-γ and stimulate the immune response. Therefore, the experiments were extended to ascertain the role of biological factors involved in stimulating IFN-γ. Under acidic conditions, HeLa cells could release exosome-anchored IL-18. IFN-γ expression in HCM-stimulated Jurkat and THP-1 cells can be boosted by exosome-anchored IL-18. These findings suggest that cancer cells might stimulate the immune response under acidic conditions by upregulating the IFN-γ associated with IL-18. Furthermore, the nuclear localization of NF-κB in Jurkat and THP-1 cells cultured in HCM (pH 6.9, pH 6.5) medium versus HCM (pH 7.4) medium suggests an involvement of IL-18 and NF-κB mediated pathways in IFN-γ stimulated cell survival.

Further attempts were made to investigate how cancer cells could escape the IFN-γ mediated attack stimulated by cancer cells via IL-18 on immune cells in an acidic environment and what role the acidic medium played as an intermediary medium between the interactions of cancer and immune cells. Therefore, a cytotoxic effect assessment after the reversal of pH was performed. Results revealed that reversal and retaining of the pH to 7.4 improved the cytotoxic effect of the immune cells. This study suggests that, while cancer cells could stimulate IFN-γ expression in immune cells, the efficiency of IFN-γ in an acidic environment was compromised. Overall, it appeared that the acidic microenvironment around the tumor and immune system could interfere with immune response, and reversal of this tumor microenvironment could be a novel approach to enhance immunotherapy and treat the tumor. Similarly, a recent study revealed that an acidic environment could impair the function of immune cells, which could be reverted by blocking the acidic environment [45, 46]. Furthermore, Pilon-Thomas et al. [47] suggested that the reversal of tumor pH could also improve the potential of immune cells to fight cancer [48]. Overall, this study suggests that the acidic environment could not only restrict the immune cells’ performance by downregulating the major effector molecules like IFN-γ but also act as a barrier by modulating the various major effector molecules like IFN-γ in the extracellular environment. Hence, it contributes to immune escape. These findings agree with those of Fischer et al. [41], who discovered that acidosis impairs cytolytic activity and causes anergy in CD8^+^ T lymphocytes.

## Conclusion

According to the findings, the acidic pH around cancer cells alters the expression of cytokines like IFN-γ in immune cells. The acidic microenvironment also modulated the interaction between cancer and immune cells through IFN-γ that was regulated by IL-18 and the NF-κB mediated pathway. Besides, it could also restrict the immune response by acting as an intermediate modulating medium resulting in immune escape. Therefore, managing the acidic microenvironment could provide a significant approach to anticancer and immunotherapy. Although the present study provides the preliminary evidences about the role of acidic microenvironment in cancer and immune cells interactions. The study provides evidences from in vitro models. The study must be validated in vivo models to reach specific conclusions.
